# Giant gastric gastrointestinal stromal tumor: A case report

**DOI:** 10.1016/j.ijscr.2025.111420

**Published:** 2025-05-09

**Authors:** Durga Neupane, Narendra Pandit

**Affiliations:** a Department of Surgery, B.P. Koirala Institute of Health Sciences, Dharan, Nepal; b Department of Surgical Gastroenterology, Birat Medical College Teaching Hospital (BMCTH), Biratnagar, Nepal

**Keywords:** Case report, GIST, Gastric, Surgical resection, Neoadjuvant therapy

## Abstract

**Introduction and importance:**

Gastrointestinal stromal tumor is the most common mesenchymal tumor of the digestive tract. With occurrence in <5 % of all gastrointestinal tract tumors, they encompass 60 % of all gastric stromal tumors.

**Case presentation:**

A 60-year-old man presented to the emergency department with pain abdomen and lump for the past 4 months. Contrast enhanced computed tomography (CECT) abdomen showed a large (largest dimension-25 cm), well-defined, soft tissue lesion on left side of abdominal cavity, crossing the midline to the right side. En-bloc tumor resection with wedge resection of the gastric body was performed. On histopathological examination, definitive diagnosis of giant gastric gastrointestinal stromal tumor (GIST) was established. Postoperatively, the patient received adjuvant oral Imatinib therapy, and is recurrence free at 3-years of follow-up.

**Clinical discussion:**

The clinical manifestations of GIST are non-specific and vary from being asymptomatic to several signs and symptoms such as abdominal pain, a palpable mass, bleeding, intestinal occlusion, and perforation. Surgical resection is the treatment of choice for GISTs, and neoadjuvant imatinib mesylate therapy for locally advanced GISTs confers good prognosis.

**Conclusions:**

Despite the fact that luminal bleeding is the most common presentation, sometimes the tumor may grow exophytically without any symptoms. If large enough as observed in the present case, they may present to emergency department as intra-tumoral bleed. Surgical resection and neoadjuvant therapy with imatinib is equally advantageous even for giant GIST. Diagnosis and therapeutical protocol of GISTs should be established by a trans-disciplinary team.

## Highlights

• Gastrointestinal stromal tumor is the most common mesenchymal tumor of the digestive tract.
• Surgical resection is treatment of choice.
• Neo-adjuvant chemotherapy is effective.
• On immunohistochemistry, the tumor stained positive for CD-117, CD34 and DOG-1.

## 1 Introduction

Gastrointestinal stromal tumor (GIST) is the most common tumor of the digestive tract, mesenchymal in origin. With occurrence in <5 % of all gastrointestinal tract tumors, they encompass 60 % of all gastric stromal tumors [1]. Their development occurs from intestinal cells of Cajal as a result of mutation of KIT and platelet-derived growth factor receptor alpha (PDGFRA) [2,3]. Because these tumors often do not present with characteristic symptoms, their diagnosis is often hindered. The size of giant GISTs of the stomach are usually >10 cm in diameter, and are accidentally detected by presentation of delayed symptoms, such as abdominal pain, anemia due to digestive bleeding, or a palpable lump [4]. Surgical resection is the treatment of choice for GISTs, and neoadjuvant imatinib mesylate therapy for locally advanced GISTs confers good prognosis [5,6]. This case report has been reported in line with the SCARE criteria [7].

## 2 Case presentation

**Fig. 1:**
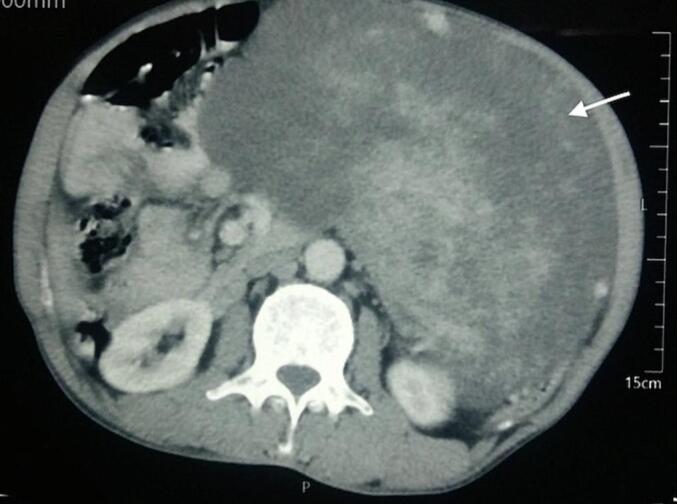
Contrast-enhanced CT abdomen showing a large, well-defined, mixed density tumor occupying the left side of the abdomen without distinct origin (arrow).

**Fig. 2:**
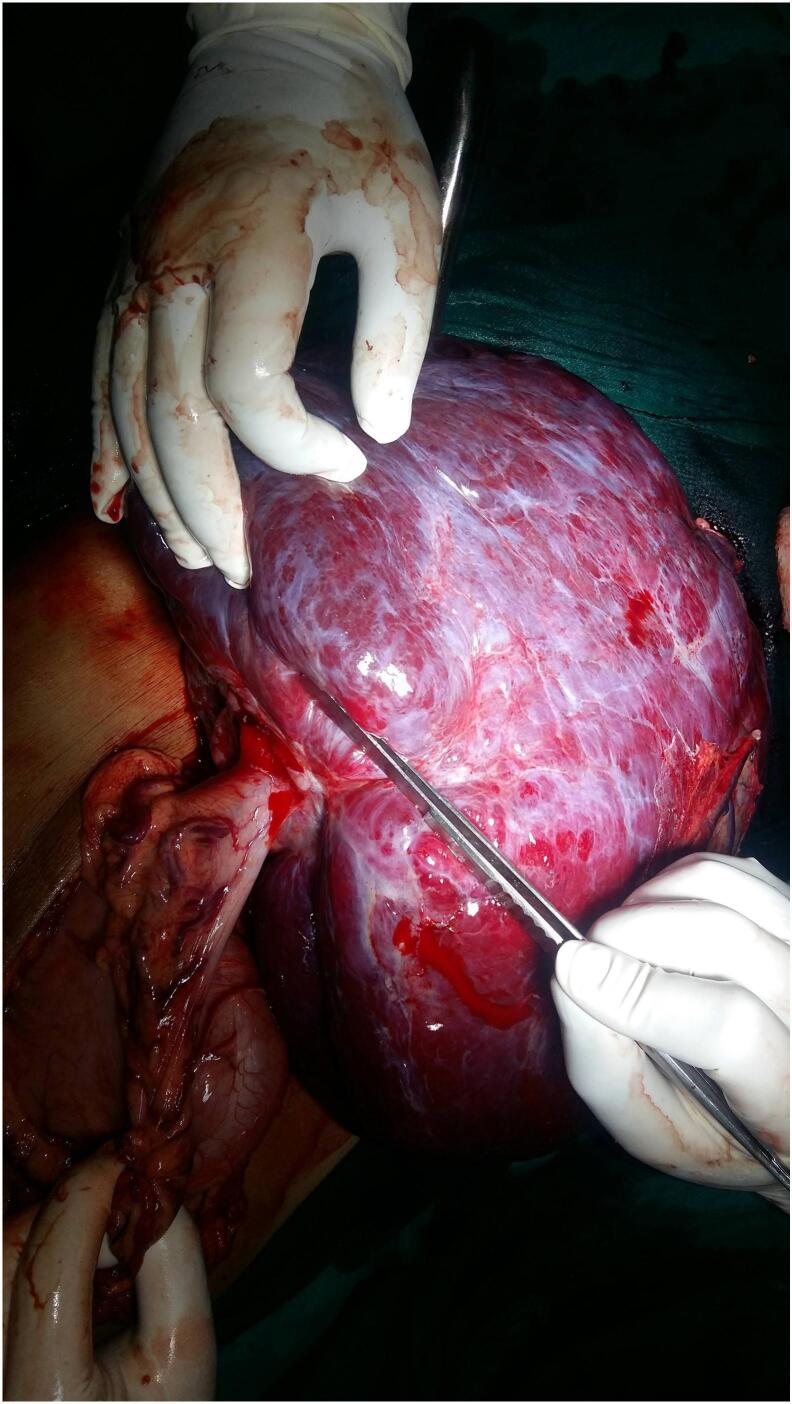
Intra-operative image showing reddish, soft cystic tumor (intra-tumoral bleed) with pedunculated attachment to the body of stomach.

A 60-year-old man presented to the emergency department with abdominal pain and lump for the past 4 months. The pain had suddenly increased in intensity just before his presentation. The patient denied any luminal bleeding, vomiting, anorexia or weight loss. He was hemodynamically stable, despite anemia (Hemoglobin- 7.0 g/dl). Contrast enhanced computed tomography (CECT) abdomen showed a large (largest dimension-25 cm), well-defined, soft tissue lesion on left side of abdominal cavity, crossing the midline to the right side (Fig. 1). It has a heterogeneous solid enhancing and fluid density components abutting left kidney and aorta posteriorly and displacing bowel loops towards the right side suggestive of soft tissue tumor. Fig. 2 shows an intra-operative image showing reddish, soft cystic tumor (intra-tumoral bleed) with pedunculated attachment to the body of stomach. En-bloc tumor resection with wedge resection of the gastric body was performed. On histopathological examination, definitive diagnosis of giant GIST was established. On microscopy, the tumor showed spindle shaped cells admixed with epithelioid component. There was presence of necrosis with mitotic figure of >10/50 high power field. On immunohistochemistry, the tumor stained positive for CD-117, CD34 and DOG-1. Postoperatively, the patient received adjuvant oral Imatinib therapy, and is recurrence free at 3-years of follow-up.

## 3 Discussion

GISTs representing 1–3 % of the tumors of the gastrointestinal, are mostly diagnosed in the stomach, and more frequent in adults [8]. According to the Fletcher's criteria, GIST is considered to be malignant based on the following characteristics: tumor size >20 mm, presence of large haemorrhagic and necrotic areas, high mitotic rate (>20 mitoses at 50 HPF), and high Ki67 index [9].

GISTs often have solid tumor components. Cystic components of GISTs are rare. GISTs with cystic changes are more frequently encountered in high-grade malignancies. The formation of large cystic spaces is due to aggressive tumor growth and lack of adequate blood supply, necrosis, haemorrhage, liquefaction, and cystic degeneration [10].

The clinical manifestations of GIST are non-specific and vary from being asymptomatic to several signs and symptoms such as abdominal pain, a palpable mass, bleeding, intestinal occlusion, and perforation. The diagnosis of GIST of the stomach is frequently not clear preoperatively, as evident in our case. GISTs of the stomach with cystic changes are uncommon and often misdiagnosed as hepatic or pancreatic lesions [11,12]. Imaging study and FNAC could be helpful. The preoperative diagnosis of cystic GIST is typically made using imaging studies such as CT or MRI, which reveal a cystic or mixed mass originating from the gastrointestinal tract. Endoscopic ultrasound (EUS) can further characterize the lesion and guide fine-needle aspiration (FNA) for cytology. Immunohistochemical staining for markers like CD117 and DOG1 on FNA samples helps confirm the diagnosis. In cases where FNA is inconclusive, a core needle biopsy or surgical resection may be necessary for definitive diagnosis.

CD117 is positive in 90 % of GISTs. The tumor tissue specimen in our case was also intensely positive for CD117. Our patient was also positive for DOG1, another sensitive marker that is positive in 95 % of the cases [12], and positive for CD34, which is positive in 50 to 80 % of the cases [12].

GISTs often have malignant potential and are highly invasive and tend to metastasize to remote organs [13]. Metastases of GIST commonly occur in the abdominal cavity and liver, and rarely in the lymph nodes. Though necrosis and cystic degeneration were observed in the tumor specimen of our patient, no metastasis to lymph nodes and remote organs was observed.

Wang et al. reported seven cases of GIST with cystic changes and they did not find recurrence 9 to 80 months following surgical resection [14]. In line with the aforementioned, no recurrence was observed in our case at a median follow-up of 3 years.

Surgical resection is the treatment of choice for GISTs, and neoadjuvant imatinib mesylate therapy for locally advanced GISTs confers good prognosis [5,6]. En-bloc tumor resection with wedge resection of the gastric body was performed in our case. GISTs of the stomach with cystic changes are rare and may defy diagnosis preoperatively. Our case emphasizes that GIST with cystic changes should be considered in the differential diagnoses of hepatic and pancreatic lesions. Additionally, immunohistochemistry with CD117, DOG1, and other molecular markers is crucial for diagnosis of GIST of the stomach and facilitates optimal treatments for GIST.

## 4 Conclusions

We would like to deliver following recommendation. Despite the fact that luminal bleeding is the most common presentation, sometimes the tumor may grow exophytically without any symptoms. If large enough as observed in the present case, they may present to emergency department as intra-tumoral bleed. Surgical resection and neoadjuvant therapy with imatinib is equally advantageous even for giant GIST. Diagnosis and therapeutical protocol of GISTs should be established by a trans-disciplinary team and to be adapted for the clinicopathological and molecular profile of each patient to confer a good prognosis.

## Authors' contribution

Both the authors contributed equally to writing and preparing the manuscript. The final version of the article is approved by both authors.

## Consent for publication

Written informed consent was obtained from the patient for publication of this case report and accompanying images. A copy of the written consent is available for review by the Editor-in-Chief of this journal on request.

## Ethical approval

Ethical approval is waived for publishing case report by our institution.

## Guarantor

Narendra Pandit.

## Provenance and peer review

Not commissioned, externally peer reviewed.

## Funding sources

None.

## Research registration

No new surgical techniques or new equipment/technology was used.

## Declaration of competing interest

None.
